# Supplementary material for: Chromothripsis during telomere crisis is independent of NHEJ, and consistent with a replicative origin
Source: Genome Res. 2019 May;29(5):737–49. doi: 10.1101/gr.240705.118 (PMC6499312; doi:10.1101/gr.240705.118)
Supplement: Supplemental Material [file supp_gr.240705.118_Supplemental_file_1.zip › contigs/annotated_contigs/DB37/contig.2.DB37_length_449_mean_cov_6.01336302895.docx]

**DB37_length_449_mean_cov_6.01336302895**

```
CATAGCAAGAACTTTCTTATGACATAATGCAAAATAAAGAACTAAAGACATTCTACTCTTTGTTCCATTCTAGTGTTATAACCTAAATT
 >chr1:193063720-193063886 + E=2e-88
ATCACATGGGAATGAAATTCTTGTCTAAATTCTTGATCAAGAGGTTTCCTAATGGCAGTTTAAATCCAAGTAAGA|AG|AGAAGATTAA
                                                                            >chr1:19320
ATACTGATTTGCATAAAATTGCCAAATAAAATCTTTACAAATTTAGAATAAGTTTAAGCTGATATCTGGATATATTTTAGATATGTTAT
8593-193208878 + E=4e-160
ACTTGACGTGGCTATATTTATTTGTTGGTTGACATTTAAAATTTTTTTTTGTTGATACATAATATTTGTACATATTTATGGTGTACATG

TGATATTTTGTTACATGGATAGAATGTGTAATGATCAAGTCAGTATTTAGGGTATGTATCACCTTGAGTGTTTATTATTTCTGTGTTTT

GGGAAC
```
